# Potential tolerability of ancient grains in non-celiac wheat sensitivity patients: A preliminary evaluation

**DOI:** 10.3389/fmed.2022.995019

**Published:** 2022-09-28

**Authors:** Aurelio Seidita, Pasquale Mansueto, Alessandra Giuliano, Marta Chiavetta, Francesca Mandreucci, Maurizio Soresi, Mattia Pistone, Stella Compagnoni, Daniele Castellucci, Gianluca Bisso, Francesco Faraci, Salvatore Maestri, Rosaria Disclafani, Anna Sapone, Alessio Fasano, Antonio Carroccio

**Affiliations:** ^1^Unit of Internal Medicine, Department of Health Promotion Sciences, Maternal and Infant Care, Internal Medicine and Medical Specialties (PROMISE), University of Palermo, Palermo, Italy; ^2^Unit of Internal Medicine, “V. Cervello” Hospital, Ospedali Riuniti “Villa Sofia-Cervello”, Palermo, Italy; ^3^Department of Health Promotion Sciences, Maternal and Infant Care, Internal Medicine and Medical Specialties (PROMISE), University of Palermo, Palermo, Italy; ^4^Istituto Zooprofilattico (IZSS), Palermo, Italy; ^5^Division of Pediatric Gastroenterology and Nutrition, Mucosal Immunology and Biology Research Center, Center for Celiac Research, Harvard Medical School, Massachusetts General Hospital for Children, Boston, MA, United States

**Keywords:** Non-celiac Wheat Sensitivity (NCWS), ancient grains, wheat free diet, wheat tolerability, Amylase-Trypsin Inhibitors (ATIs)

## Abstract

**Background and aims:**

A wheat-free diet (WFD) represents the elective treatment for Non-celiac Wheat Sensitivity (NCWS) patients. Preliminary reports have shown a possible better tolerability of ancient grains in these subjects. The aim of this observational study was to evaluate the frequency of consumption of ancient grains and its correlation with clinical manifestations in NCWS patients.

**Methods:**

223 NCWS patients were recruited, and their consumption of ancient grains was monitored. Participants were first administered a modified version of the Pavia/Biagi questionnaire to investigate their adherence to “modern WFD.” The appearance/exacerbation of symptoms after ingestion of ancient grains was then assessed with WHO toxicity grading scale.

**Results:**

50.2% of the recruited patients reported consuming ancient grains before NCWS diagnosis; the diagnostic delay in this group was significantly higher than in non-consumers [median (range) 72 (6–612) vs. 60 months (3–684), *P* = 0.03] and these patients reported lower frequency of constipation (*P* = 0.04). Of the 107 patients with optimal adherence to modern WFD, 14 reported eating ancient wheat after NCWS diagnosis. Among them, 5 reported milder symptoms than those caused by modern wheat intake and 3 had an excellent tolerability without symptoms. Timilia/Tumminia variety was the most frequently used ancient grain.

**Conclusions:**

NCWS patients who consume ancient grains may receive a late diagnosis due to the possible clinical benefit (tolerability) obtained with these grains. Even after diagnosis, 10% of the patients still consumed ancient grains and had mild or no symptoms. Further studies are required to define the pathophysiological mechanism behind their putative greater tolerability.

## Introduction

The consumption of gluten- and wheat-free foods, even without a diagnosis of celiac disease (CD) or wheat allergy (WA), has greatly increased in recent years. Indeed, more and more people are self-reporting intestinal symptoms [i.e., irritable bowel syndrome (IBS)-like symptoms, such as abdominal pain, diarrhea, constipation, abdominal bloating, meteorism] and extra-intestinal manifestations (headaches, dermatitis, fatigue, etc.) secondary to wheat ingestion and, at the same time, referring the clinical benefits obtained when avoiding gluten- and wheat-containing products. Starting from this self-reported condition, about 10 years ago a panel of experts defined a new gluten-related disease, which was labeled at first as “Non-celiac Gluten Sensitivity” (1), and, subsequently, “Non-celiac Wheat Sensitivity” (NCWS) (2).

In fact, NCWS is unlike CD, where there is a clear autoimmune condition characterized by a specific serological and histological profile triggered by gluten ingestion in genetically predisposed individuals (HLA-DQ2/DQ8 positivity and non-HLA genes) (3), and it has not yet been established whether or not gluten is the real trigger in NCWS. Some authors have also suggested a role for Fermentable Oligosaccharides, Disaccharides, Monosaccharides and Polyols (FODMAPs) (4, 5) while other studies have focused their attention on the activation of both innate and acquired immunity in inducing symptoms (6, 7). In this context, it has been highlighted that wheat contains Amylase-Trypsin Inhibitors (ATIs), proteins able to activate innate immunity *via* Toll-like receptor 4 (TLR4) on myeloid cells (8). The epidemiology of NCWS is difficult to estimate because of the absence of specific biological markers. However, in tertiary centers, its frequency seems 3/6-fold that of CD, and women aged between 20 and 50 years are most affected (F:M = 6:1) (9, 10).

The pathogenesis of IBS, on the other hand, is heterogeneous; traditionally, focus has been on abnormalities in motility, visceral sensation, brain-gut interaction, and psychosocial distress. More recently, alterations in gut immune activation, intestinal permeability, visceral hypersensitivity and the intestinal and colonic microbiota have been identified in some IBS patients (11–16).

Preliminary *in vitro* and *in vivo* data suggest that the consumption of ancient grain varieties could be better tolerated by NCWS and IBS patients (17–19), thus, making it plausible that other wheat components, different from gluten, may play a leading role in NCWS pathogenesis. However, it is necessary to underline that these ancient grains are typically consumed as whole grain products, thus it cannot be ruled out that the possible benefits could be due to this practice rather than to their intrinsic composition (20). Moreover, leavened breads made with ancient wheat are usually produced with natural yeast, which contains Lactobacilli that degrade ATIs (21).

The purpose of this study was to evaluate the frequency of consumption of ancient grains and its correlation with clinical manifestations in a population of NCWS patients diagnosed by double-blind placebo-controlled challenges (DBPCCs) with modern wheat.

## Materials and methods

Our observational study consisted of a retrospective and a prospective part. In the first, retrospective part, a large group of 230 patients with NCWS, diagnosed by DBPCC with modern wheat in 3 clinical departments of Internal Medicine (“P. Giaccone” University Hospital, Palermo, Italy, “V. Cervello” Hospital, Palermo, Italy, and “Giovanni Paolo II” Hospital, Sciacca, Italy), between January 2011 and December 2020, were included. All the data from our medical records were reviewed and collected in a digital database.

In the second, prospective part of the study, all the included NCWS patients were contacted by experienced physicians between November 2021 and April 2022, at a median time of 24 months after the first recruitment, and invited to answer a questionnaire to investigate their current adherence to the modern wheat-free diet (WFD), their well-being and health conditions, their consumption of ancient wheat (before and/or after NCWS diagnosis), and the kind of ancient wheat consumed.

### Inclusion criteria

Inclusion criteria for the NCWS patients were: (A) age between 18 and 65 years old; (B) reported intestinal and/or extra-intestinal wheat-related symptoms; (C) negative serum anti-tissue transglutaminase (anti-tTG), Immunoglobulin (Ig)A and IgG antibodies; (D) absence of duodenal villous atrophy, sampled in all patients with the DQ2 or DQ8 HLA haplotypes, and assessed when they had a minimum intake of 100 grams of pasta and/or bread per day for at least 45 days or when considered clinically appropriate; (E) exclusion of WA, diagnosed by negative skin prick tests and/or serum specific IgE for wheat, gluten and gliadin detection; (F) resolution of symptoms on a strict standard elimination diet (i.e., oligoantigenic diet, without wheat, cow’s milk, egg, tomato, chocolate, and other foods reported as causing symptoms by the patients themselves), lasting at least 4 weeks, and followed by the reappearance of the same symptoms after DBPCCs with modern wheat (for details of the elimination diet and challenge methods see Supplementary File 1); (G) follow-up duration longer than 12 months after the initial diagnosis, with at least 2 outpatient visits during this period; (H) complete medical records.

### Exclusion criteria

The exclusion criteria were: (A) self-exclusion of gluten/wheat from the diet and refusal to reintroduce it for diagnostic purposes before entering the study; (B) anti-endomysium antibody (EmA) positivity in the culture medium of duodenal mucosa samples (EMA-biopsy), even in the presence of a normal villus/crypt ratio in the mucosa; (C) incomplete clinical records; (D) pregnancy; (E) abuse of alcohol and/or drugs; (F) treatment with corticosteroids and/or NSAIDs in the 2 weeks prior to duodenal biopsies (when performed); (G) diagnosis of chronic inflammatory bowel disease or other organic pathologies affecting the digestive system; (H) coexisting infectious diseases.

### Outcome measures

For each patient, the following data were extracted from the medical records and then analyzed: gender, age at diagnosis, diagnostic delay, body mass index (BMI), IBS-like symptoms presence and subtypes (diarrhea, constipation or mixed bowel movements), dyspepsia, body weight loss (established by an at least 10% reduction in body weight in 6 months or less), presence and kind of extra-intestinal symptoms, presence of autoimmune diseases, coexistence of other atopic diseases (allergic rhino-conjunctivitis and/or allergic asthma and/or atopic dermatitis), and any other comorbidities.

Adherence to the modern WFD was assessed according to a modified version of the “Pavia” or “Biagi” score (22).

In detail, the following score was used: 0 = no adherence to the modern WFD; 1–2 = poor adherence to the modern WFD; 3–4 = “strict” adherence to the modern WFD (for details, see Supplementary File 2). The score was applied to the consumption/avoidance of modern wheat; the contemporary consumption of pasta or bakery products made with ancient wheat flour was not considered a “transgression.” Patients were then asked about the specific ancient wheat flours they consumed: Perciasacchi, Senatore Cappelli, Timilia/Tumminia, Russello, Khorasan (hereafter referred to as “Kamut^®^,” as it is the commercial name best known to our patients), spelt, or others. The first four above-mentioned wheat varieties are among the most frequently cultivated and consumed ancient varieties in Sicily. The frequency of ancient wheat flour consumption was graded as frequent (>3 times per week, 50 grams at least), moderate (1–2 times per week) or rare (<1 time per week). The clinical tolerability of ancient grains was assessed according to the World Health Organization toxicity grading scale (23, 24) and both intestinal (abdominal pain, altered bowel movements, nausea/vomiting, etc.) and extra-intestinal symptoms (headache, fatigue, myalgia, etc.) were investigated.

In detail, the following grading scale was used: 0 = absence of symptoms; 1 = mild symptoms; 2 = moderate symptoms, 3 = severe symptoms, and 4 = life threatening.

### Statistical analysis

Data were expressed as mean ± standard deviation (SD) when the distribution was Gaussian, and Student’s *t* test was used to evaluate differences between group means. Comparisons between more than 2 groups were performed by ANOVA, followed by a *post hoc* analysis using the Bonferroni tests. Otherwise, data were expressed as median and range and analyzed with the Kruskal–Wallis and Mann–Whitney *U* tests. The χ^2^ test and Fisher’s exact test were used to compare the frequency values in the various population groups. The McNemar test for paired data was performed to analyze the frequency of changes in symptoms (IBS-like, dyspepsia and extra-intestinal) before and after modern WFD, according to adherence: “poor adherence” and “strict adherence.”

The study was registered on clinicaltrials.gov (registration number n. NCT03024775) and approved by the Ethics Committee of the AOUP “P. Giaccone” hospital of Palermo. Recruited patients gave their informed consent to the study.

## Results

Of the initial 230 NCWS patients, 4 were excluded due to the presence of other gastrointestinal diseases at the time of the questionnaire, and another 3 patients were excluded because they refused to answer the questionnaire. Thus, data from 223 patients were analyzed.

Table 1 shows the demographic and clinical features of the NCWS patients. Most of the patients were female (89.3%), aged between the third and fourth decade of life, and with a significant diagnostic delay following the onset of symptoms [median and range 60 (3–684) months].

**TABLE 1:** Demographic and clinical features of NCWS patients.

	NCWS (*n* = 223) (%)
**Sex** Females Males	199 (89.3) 24 (10.8)
Age at diagnosis (years, mean ± SD)	38.3 ± 12.4
Diagnostic delay (months, median and range)	60 (3–684)
**BMI (Kg/m^2^)** < 18.5 (underweight) 18.5–24.9 (normal weight) 25.0–29.9 (overweight) ≥ 30.0 (obesity)	18 (8.1) 131 (58.7) 41 (18.4) 33 (14.8)
**IBS-like symptoms presence and subtypes** None IBS-Diarrhea IBS-Constipation IBS-Mixed	28 (12.6) 109 (48.9) 32 (14.3) 54 (24.2)
Dyspepsia	140 (62.8)
Body weight loss	68 (30.5)
Extraintestinal symptoms	153 (68.6)
Neuropsychiatric symptoms	105 (47.1)
AD	70 (31.4)
Hashimoto thyroiditis	47 (21.1)
Other AD than Hashimoto thyroiditis	34 (15.2)
Coexistent atopic disease	62 (27.8)
Other comorbidities	198 (88.8)

AD, Autoimmune disease; BMI, Body Mass Index; IBS, Irritable Bowel Syndrome; NCWS, Non-Celiac Wheat Sensitivity; SD, Standard Deviation.

**FIGURE 1:**
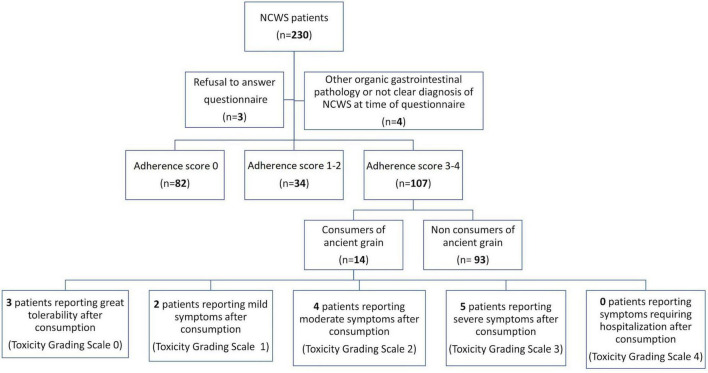
Flow-chart of the study, showing patient adherence to a modern wheat-free diet and consumption of ancient grains after Non-celiac Wheat Sensitivity (NCWS) diagnosis.

When contacted, 82 (36.9%) patients were no longer following the modern WFD (Score 0), 34 (15.2%) had poor adherence to the modern WFD (Score 1–2), and 107 (48.0%) had a Score of 3–4, which represents a strict adherence to the modern WFD (Figure 1).

The patients were then interviewed about the persistence of symptoms, which was inversely correlated with the adherence score, and NCWS patients on a strict modern WFD reported a very high frequency of symptom disappearance. Of note, even poor adherence to the modern-WFD was able to improve both IBS-like (*P* < 0.02 vs. *P* < 0.0001, respectively, in poor-adherents and strict-adherents) and extraintestinal (*P* < 0.0005 vs. *P* < 0.0001, respectively, in poor-adherents and strict-adherents) symptoms in a part of the NCWS subjects, albeit with a lower significance than strict adherence; this was not proved for dyspepsia (*P* = 0.15 vs. *P* < 0.0001, respectively, in poor-adherents and strict-adherents).

**FIGURE 2:**
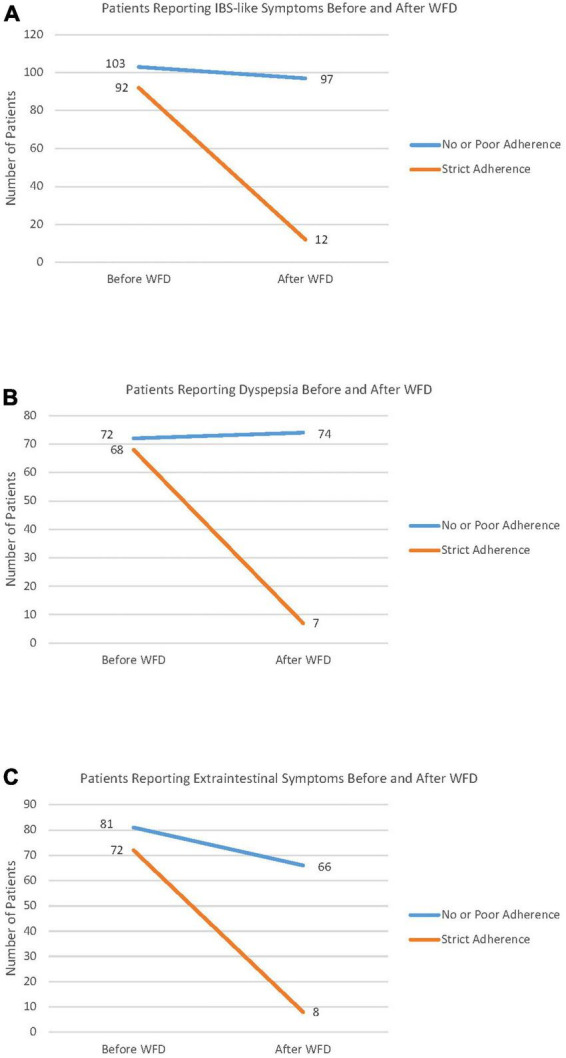
Symptom changes before and after the wheat-free diet (WFD) according to a modern WFD adherence score. Patients with an adherence score between 0 and 2 were classified as non- or poorly adherent to the wheat-free diet. Patients with an adherence score of 3–4 were considered as strictly adherent to the diet. Panel **(A)** shows the number of patients reporting Irritable Bowel Syndrome-like (IBS) symptoms, before and after the WFD. Panel **(B)** shows the number of patients reporting dyspepsia, before and after WFD. Panel **(C)** shows the number of patients reporting extraintestinal symptoms, before and after WFD.

Figure 2 shows the number of patients reporting IBS-like symptoms (Figure 2A), dyspepsia (Figure 2B) and extra-intestinal symptoms (Figure 2C) before and after the WFD, according to the degree of adherence to this diet. In the group reporting strict adherence to the modern WFD, the frequency of IBS-like symptom disappearance was significantly higher than in the NCWS subjects with no or poor adherence to the modern-WFD (87.0% vs. 5.8%; *P* < 0.0001), and this also applied to dyspepsia (89.7% vs. 0.0%; *P* < 0.0001) and extraintestinal symptoms (88.9% vs. 18.5%; *P* < 0.0001). Table 2 shows the demographic and clinical features of the 223 NCWS patients according to ancient grain consumption before NCWS diagnosis 112 (50.2%) patients reported habitual consumption of ancient grains before NCWS diagnosis. A higher diagnostic delay was observed in these patients compared to those who had never consumed ancient grain products [72 (6–612) months vs. 60 months (3–684), *P* = 0.03]; moreover, a higher frequency of constipation was observed in the patients who had never consumed ancient wheat (19.8% vs. 8.9%, *P* = 0.04). No other statistically significant differences were demonstrated.

**TABLE 2:** Demographic and clinical features of NCWS patients according to ancient grain consumption before NCWS diagnosis.

	Non-consumers (*n* = 111) (%)	Consumers (*n* = 112) (%)	*P*
**Sex** Female Male	98 (88.3) 13 (11.7)	101 (90.2) 11 (9.8)	NS NS
Age at diagnosis (years, mean ± SD)	38.5 ± 12.4	38.2 ± 12.6	NS
Diagnostic delay (months, median and range)	60 (3–684)	72 (6–612)	0.03
**BMI (Kg/m^2^)** < 18.5 (underweight) 18.5–24.9 (normal weight) 25.0–29.9 (overweight) ≥ 30.0 (obesity)	7 (6.3) 68 (61.3) 17 (15.3) 19 (17.1)	11 (9.9) 63 (56.2) 24 (21.4) 14 (12.5)	NS NS NS NS
**IBS-like symptoms presence and subtypes**			
None	13 (11.7)	15 (13.4)	NS
IBS-Diarrhea	55 (49.5)	54 (48.2)	NS
IBS-Constipation	22 (19.8)	10 (8.9)	0.04
IBS-Mixed	25 (22.5)	29 (25.9)	NS
Dyspepsia	68 (61.3)	72 (64.3)	NS
Body weight loss	35 (31.5)	33 (29.5)	NS
Extraintestinal symptoms	76 (68.5)	77 (68.8)	NS
Neuropsychiatric symptoms	49 (44.1)	56 (50.0)	NS
AD	37 (33.3)	33 (29.5)	NS
Hashimoto’s thyroiditis	26 (23.4)	21 (18.6)	NS
Other AD than Hashimoto’s thyroiditis	18 (16.2)	16 (14.3)	NS
Coexistent atopic disease	31 (27.9)	31 (27.7)	NS
Comorbidity	101 (91.0)	97 (86.6)	NS

AD, Autoimmune disease; BMI, Body Mass Index; IBS, Irritable Bowel Syndrome; NCWS, Non-celiac Wheat Sensitivity; SD, Standard Deviation.

When contacted for the prospective study, in the subset of patients who declared a strict adherence to the modern WFD (107 subjects), 14 (13.1%) reported consuming ancient wheat at least once after NCWS diagnosis. Their individual data are shown in Table 3. Three patients reported excellent tolerability (toxicity grading scale 0), and grain consumption was in the form of whole grain products. Furthermore, 2 patients had mild symptoms (toxicity grading scale 1), 4 had moderate symptoms (toxicity grading scale 2), and 5 patients had severe symptoms (toxicity grading scale 3) after consuming products based on either refined or whole ancient grains. Among the 11 consumers of ancient wheat with consequent symptoms, 5 (45.5%) defined symptoms as more tolerable than those caused by eating modern grain products. Overall, the NCWS patients with adherence score 3–4 tried ancient grain products after a median time of 24 (range 1–48) months on a strict WFD. As regards the reason for consuming ancient wheats, patients reported their better palatability and lower cost compared to wheat-free products.

**TABLE 3:** Tolerability and consumption of ancient grains after NCWS diagnosis in 14 patients with strict adherence to modern WFD, according to toxicity grading scale order.

Patient’s initials	Toxicity Grading Scale	Frequency of consumption	Time from diagnosis to starting consumption of ancient grains (months)	Kind of ancient grain consumed	Whole or refined products	Intestinal and/or extraintestinal symptoms	Are the symptoms caused/triggered by ancient grains more tolerable than those caused/triggered by modern grains?
M.C.	0	Frequent	48	Russello grain	Whole	No	NA
C.S.	0	Frequent	1	Perciasacchi, Senatore Cappelli, Timilia/Tumminia, Russello	Whole	No	NA
R.A.	0	Moderate	1	Perciasacchi, Senatore Cappelli, Timilia/Tumminia, Russello, Kamut^®^, spelt	Whole	No	NA
G.G.	1	Moderate	24	Perciasacchi, Timilia/Tumminia	Refined	Both	Yes
A.V.	1	Rare	6	Perciasacchi, Timilia/Tumminia, Russello, Kamut^®^, spelt	Whole	Both	Yes
R.D.	2	Rare	48	Perciasacchi, Senatore Cappelli	Refined	Both	Yes
M.M.	2	Frequent	24	Perciasacchi, Senatore Cappelli, Timilia/Tumminia	Whole	Both	Yes
L.O.	2	Rare	24	Timilia/Tumminia	Refined	Intestinal	No
B.S.	2	Rare	0	Kamut^®^	Refined	Intestinal	Yes
G.T.	3	Moderate	24	Perciasacchi, Senatore Cappelli, Timilia/Tumminia, Russello, Kamut^®^, spelt	Whole	Both	No
T.C.	3	Rare	3	Timilia/Tumminia, Kamut^®^	Refined	Both	No
F.R.	3	Rare	96	Timilia/Tumminia, Kamut^®^	Refined	Both	No
L.I.	3	Rare	24	Spelt	Refined	Both	No
D.R.	3	Moderate	48	Timilia/Tumminia	Whole	Both	No

NA, Not Applicable; NCWS, Non-celiac Wheat Sensitivity; WFD, Wheat-Free Diet.

**FIGURE 3:**
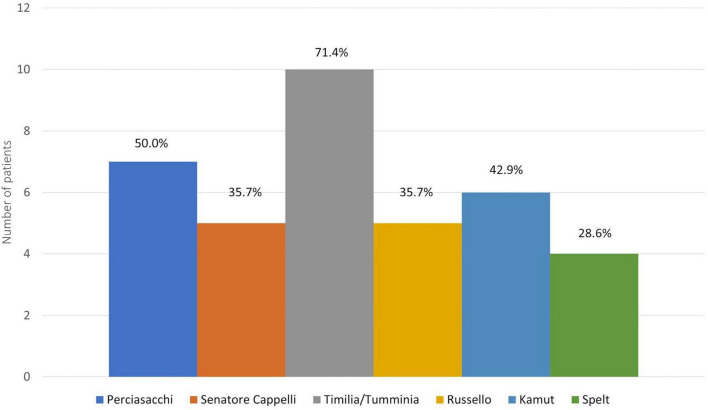
Kind of ancient grains consumed by patients with strict adherence to the modern wheat-free diet.

Finally, Figure 3 shows the kinds of ancient grains consumed by the NCWS patients with adherence score 3–4: Timilia/Tumminia (71.4%), Perciasacchi (50.0%), and Kamut^®^ (42.9%) were the most frequent.

## Discussion

Treatment of NCWS essentially consists of prescribing a WFD, but due to the problematical nutritional, economic, social and psychological implications of adopting a classic WFD, there is growing interest in finding other viable options suitable for NCWS sufferers. For many people, and according to some preliminary scientific reports, the most plausible alternative would seem to be a diet that simply replaces “modern” with “ancient” grains (25). However, no studies to date have evaluated the real frequency of ancient wheat variety consumption in NCWS patients diagnosed by the rigorous DBPCC method, or the putative higher tolerability of these wheats in this wheat-related disease.

In our study, we evaluated 223 NCWS patients, diagnosed by DBPCC, to assess the frequency of a dietary intake of ancient grains, before and/or after diagnosis, and the possible effects on NCWS-related symptoms.

The demographic and clinical characteristics of the recruited NCWS patients are in agreement with existing reports in the scientific literature and with our own previous studies (26–32). Our patients showed a good, but not very high percentage of strict adherence to the modern WFD (48.0%), likely reflecting a strong self-perceived relationship between the recurrence of symptoms and the intake of modern wheat, and their attenuation or disappearance thanks to the elimination diet. As a matter of fact, all our patients with adherence score 3–4 reported a strong reduction or a complete disappearance of symptoms when following the modern WFD. It is interesting to note that in our study group even the patients with poor adherence to the diet (Score 1–2) reported a significant improvement in IBS-like and extra-intestinal symptoms in comparison to the period before the NCWS diagnosis. Nevertheless, about one third of all the patients had stopped following the WFD.

Regarding the consumption of ancient grains, 50.2% of our patients reported that they had habitually consumed ancient grains before NCWS diagnosis. A greater diagnostic delay was statistically more frequent in these subjects, a finding which could be the result of a putative clinical benefit obtained, which could have led patients to postpone the “diagnostic contact” with physicians. In addition, the lower frequency of constipation in these patients is interesting, and it could be linked to the very frequent use of ancient wheat flours in the form of whole grain products, thus with a higher fiber content compared to modern ones, which are much more frequently used refined. However, the consumption of additional vegetable fibers and water intake (which might influence bowel movements) was not investigated.

To assess the potential tolerability of ancient grains in NCWS patients after diagnosis, we evaluated only the recruited patients with adherence scores 3–4, thus eliminating any modern wheat intake effects. In this way, we selected only patients that had completely eliminated modern wheat from their diet and were, therefore, clinically completely well. In this subset of patients, the percentage of ancient grain consumers was 13.1% (14 patients). Despite being aware that they were breaking the WFD by consuming ancient grains, these patients reported that they had done so to evaluate their tolerability of the ancient grains because of the greater palatability they offered, and the lower cost compared to wheat-free products. The decision to try ancient grains was made after a median period of 24 months following NCWS diagnosis and in conditions of well-being. Only 3 patients reported excellent tolerability (toxicity grading scale 0). Among the patients who developed symptoms, 45.5% reported more tolerable symptoms than those secondary to modern wheat intake, and 36% consumed ancient grains more than once a week. As regards the various grain varieties consumed, we recorded a high consumption of Kamut^®^ (42.9%) and of ancient Sicilian varieties, in particular Timilia/Tumminia (71.4%) and Perciasacchi (50.0%). In addition, all patients with a toxicity grading scale 0 ate wholegrain products, while 63.6% of patients with toxicity grading scale 1–3 consumed refined ones.

No definitive conclusions can be drawn from this study due to the small sample population of NCWS patients consuming ancient grains. Moreover, tolerability to the intake of ancient grains in these patients was variable, probably as a result of the different varieties of ancient grains consumed and the use of either whole grain or refined products. Nevertheless, the present study integrates findings already reported in the literature and confirms the need to further investigate the pathophysiology of NCWS, as it would appear to indicate that gluten is not the only “culprit.” Indeed, some data, mainly *in vitro*, suggest that a fair percentage of patients with NCWS could better tolerate certain varieties of ancient grains (17, 18). According to other authors, the symptoms in these patients might be linked not only to gluten but also to the presence of FODMAPs and/or ATIs in ingested wheat (2, 4, 5, 8, 33). It could be hypothesized that, the better tolerance to some ancient grains could be explained by the lower presence of FODMAPs and ATIs or other toxic peptides, compared to modern wheats (34–36). However, evidence in this regard seems to indicate that the amount of such components could be related to both the wheat genotype and the leavening methodology used (37, 38).

Our study has several limitations. First, the clinical data were collected at the time of diagnosis and used retrospectively. Thus, a selection bias must be considered, and our findings need to be confirmed in a larger and specifically designed prospective study. Second, the patients’ biochemical/immunological/histological response to the intake of ancient grains was not evaluated, and the self-reported consumption of ancient grains was not stratified in terms of quantity (grams). Consequently, we have no data to explain why only a certain percentage of NCWS subjects tolerated ancient wheat. Future studies should be planned to determine the biological basis of our results. Third, the NCWS patients who ate ancient wheat consumed different grains, very probably from different regional locations and grown in different seasons. Since the biochemical composition of grains is known to be influenced by these factors, we cannot extend the concept of a safe use of the tolerated grains quoted in our study without taking them into account. Moreover, it was not possible to assess any association between a specific symptom and the consumption of a certain variety of ancient wheat. Fourth, we did not investigate the reasons for non-adherence to the WFD in a percentage of the NCWS patients recruited to the study. Finally, we used a modified version of the adherence score to evaluate adherence to the “modern” WFD, which had not been previously validated for this specific purpose, but for all gluten-containing products in patients with CD.

However, the study does have some strengths: it is the first research study specifically aimed at evaluating the use of ancient grains and their clinical effects in NCWS patients. All the patients included were diagnosed by the DBPCC method, thus involving subjects identified by the current diagnostic gold standard.

In conclusion, our study shows a high frequency of consumption of ancient grains in NCWS patients before diagnosis, with a greater diagnostic delay and lower constipation frequency in this population, as well as a variable tolerability among the patients who consumed ancient grains after NCWS diagnosis. Further research is needed to define the pathophysiological mechanisms underlying their greater tolerability compared to modern grains in at least a subgroup of patients suffering from NCWS.

## Data availability statement

The datasets presented in this article are not readily available because the study presented here is a preliminary part of a larger project funded by the Italian Ministry of Health. Therefore, all, or part of, this data will be used to complete the main project. For this reason, the data cannot be provided. Requests to access the datasets should be directed to PM, pasquale.mansueto@unipa.it.

## Ethics statement

The studies involving human participants were reviewed and approved by Ethics Committee of the AOUP “P. Giaccone” Hospital of Palermo. The patients/participants provided their written informed consent to participate in this study.

## Author contributions

ASe, AC, and PM had full access to all the data in the study and take full responsibility for the integrity of the data, the accuracy of the data analysis, and conceptualized and designed the study. PM, MP, AC, SC, DC, MC, GB, FF, SM, AG, and FM contributed to the acquisition of data. ASe, PM, AG, MS, MP, RD, SC, DC, MC, GB, FF, SM, AF, ASa, and AC analyzed and interpreted the data. ASe, AG, PM, MP, AC, ASa, and AF drafted the manuscript. AC, PM, ASe, AG, ASa, and AF contributed to critical revision of the manuscript for important intellectual content. MS contributed to statistical analysis. AC and PM obtained the funding. AC and AF supervised the study. All authors contributed to the article and approved the submitted version.

## Abbreviations

anti-Ttg: anti-tissue transglutaminase antibodies
ATI: amylase/trypsin Inhibitors
BMI: body mass index
CD: celiac disease
DBPCC: double-blind placebo-controlled challenges
EmA: anti-Endomysium antibodies
FODMAPs: fermentable oligosaccharides, disaccharides, monosaccharides and polyols
HLA: human leukocyte antigens
IBS: irritable bowel syndrome
Ig: Immunoglobulin
NCWS: non-celiac wheat sensitivity
SD: standard deviation
TLR4: toll-like receptor 4
WA: wheat allergy
WFD: wheat-free diet.

## References

[1] Sapone A Bai JC Ciacci C Dolinsek J Green PH Hadjivassiliou M Spectrum of gluten-related disorders: consensus on new nomenclature and classification. *BMC Med.* (2021) 10:13. 10.1186/1741-7015-10-13 22313950 PMC3292448

[2] Carroccio A Rini G Mansueto P. Non-celiac wheat sensitivity is a more appropriate label than non-celiac gluten sensitivity. *Gastroenterology.* (2014) 146:320–1. 10.1053/j.gastro.2013.08.061 24275240

[3] Caio G Volta U Sapone A Leffler DA De Giorgio R Catassi C Celiac disease: a comprehensive current review. *BMC Med*. (2019) 17:142. 10.1186/s12916-019-1380-z 31331324 PMC6647104

[4] Biesiekierski JR Peters SL Newnham ED Rosella O Muir JG Gibson PR. No effects of gluten in patients with self-reported non-celiac gluten sensitivity after dietary reduction of fermentable, poorly absorbed, short-chain carbohydrates. *Gastroenterology.* (2013) 145:320–8.e1-3. 10.1053/j.gastro.2013.04.051 23648697

[5] Skodje GI Sarna VK Minelle IH Rolfsen KL Muir JG Gibson PR Fructan, rather than gluten, induces symptoms in patients with self-reported non-celiac gluten sensitivity. *Gastroenterology.* (2018) 154:529–39.e2. 10.1053/j.gastro.2017.10.040 29102613

[6] Brottveit M Beitnes AC Tollefsen S Bratlie JE Jahnsen FL Johansen FE Mucosal cytokine response after short-term gluten challenge in celiac disease and non-celiac gluten sensitivity. *Am J Gastroenterol.* (2013) 108:842–50. 10.1038/ajg.2013.91 23588237

[7] Schuppan D Pickert G Ashfaq-Khan M Zevallos V. Non-celiac wheat sensitivity: differential diagnosis, triggers and implications. *Best Pract Res Clin Gastroenterol*. (2015) 29:469–76. 10.1016/j.bpg.2015.04.002 26060111

[8] Zevallos VF Raker V Tenzer S Jimenez-Calvente C Ashfaq-Khan M Rüssel N Nutritional wheat amylase-trypsin inhibitors promote intestinal inflammation via activation of myeloid cells. *Gastroenterology.* (2017) 152:1100–33.e12. 10.1053/j.gastro.2016.12.006 27993525

[9] Carroccio A Giambalvo O Blasca F Iacobucci R D’Alcamo A Mansueto P. Self-reported non-celiac wheat sensitivity in high school students: demographic and clinical characteristics. *Nutrients.* (2017) 9:771. 10.3390/nu9070771 28753927 PMC5537885

[10] Volta U Bardella MT Calabrò A Troncone R Corazza GR. Study group for non-celiac gluten sensitivity. An Italian prospective multicenter survey on patients suspected of having non-celiac gluten sensitivity. *BMC Med.* (2014) 12:85. 10.1186/1741-7015-12-85 24885375 PMC4053283

[11] Simrén M Barbara G Flint HJ Spiegel BM Spiller RC Vanner S Intestinal microbiota in functional bowel disorders: a Rome Foundation report. *Gut*. (2013) 62:159–76. 10.1136/gutjnl-2012-302167 22730468 PMC3551212

[12] Dupont HL. Review article: evidence for the role of gut microbiota in irritable bowel syndrome and its potential influence on therapeutic targets. *Aliment Pharmacol Ther*. (2014) 39:1033–42. 10.1111/apt.12728 24665829

[13] Zhou Q Zhang B Verne GN. Intestinal membrane permeability and hypersensitivity in the irritable bowel syndrome. *Pain.* (2009) 146:41–6. 10.1016/j.pain.2009.06.017 19595511 PMC2763174

[14] Collins SM. A role for the gut microbiota in IBS. *Nat Rev Gastroenterol Hepatol*. (2014) 11:497–505. 10.1038/nrgastro.2014.40 24751910

[15] Farrè R Tack J. Food and symptom generation in functional gastrointestinal disorders: physiological aspects. *Am J Gastroenterol.* (2013) 108:698–706. 23458851 10.1038/ajg.2013.24

[16] Shepherd SJ Lomer MC Gibson PR. Short-chain carbohydrates and functional gastrointestinal disorders. *Am J Gastroenterol.* (2013) 108:707–17. 10.1038/ajg.2013.96 23588241

[17] Valerii MC Ricci C Spisni E Di Silvestro R De Fazio L Cavazza E Responses of peripheral blood mononucleated cells from non-celiac gluten sensitive patients to various cereal sources. *Food Chem.* (2015) 176:167–74. 10.1016/j.foodchem.2014.12.061 25624220

[18] Alvisi P De Fazio L Valerii MC Cavazza E Salerno A Lacorte D Responses of blood mononucleated cells and clinical outcome of non-celiac gluten sensitive pediatric patients to various cereal sources: a pilot study. *Int J Food Sci Nutr*. (2017) 68:1005–12. 10.1080/09637486.2017.1315058 28420279

[19] Sofi F Whittaker A Gori AM Cesari F Surrenti E Abbate R Effect of *Triticum turgidum* subsp. Turanicum wheat on irritable bowel syndrome: a double-blinded Khorasan dietary intervention trial. *Br J Nutr*. (2014) 111:1992–9. 10.1017/S000711451400018X 24521561 PMC4405706

[20] Hoevenaars F Van der Kamp JW Van den Brink W Wopereis S. Next generation health claims based on resilience: the example of whole-grain wheat. *Nutrients*. (2020) 12:2945. 10.3390/nu12102945 32992860 PMC7599623

[21] Caminero A McCarville JL Zevallos VF Pigrau M Yu XB Jury J Lactobacilli degrade wheat amylase trypsin inhibitors to reduce intestinal dysfunction induced by immunogenic wheat proteins. *Gastroenterology*. (2019) 156:2266–80. 10.1053/j.gastro.2019.02.028 30802444

[22] Biagi F Andrealli A Bianchi PI Marchese A Klersy C Corazza GR. A gluten-free diet score to evaluate dietary compliance in patients with coeliac disease. *Br J Nutr*. (2009) 102:882–7. 10.1017/S0007114509301579 19331704

[23] Zanini B Petroboni B Not T Di Toro N Villanacci V Lanzarotto F Search for atoxic cereals: a single blind, cross-over study on the safety of a single dose of *Triticum monococcum*, in patients with celiac disease. *BMC Gastroenterol*. (2013) 13:92. 10.1186/1471-230X-13-92 23706063 PMC3664588

[24] Edwards IR Biriell C. Harmonisation in pharmacovigilance. *Drug Saf*. (1994) 10:93–102. 10.2165/00002018-199410020-00001 8011183

[25] Ianiro G Rizzatti G Napoli M Matteo MV Rinninella E Mora V A durum wheat variety-based product is effective in reducing symptoms in patients with non-celiac gluten sensitivity, a double-blind randomized cross-over trial. *Nutrients*. (2019) 11:712. 10.3390/nu11040712 30934747 PMC6521061

[26] Catassi C Alaedini A Bojarski C Bonaz B Bouma G Carroccio A The overlapping area of non-celiac gluten sensitivity (NCGS) and wheat-sensitive irritable bowel syndrome (IBS): an update. *Nutrients*. (2017) 9:1268. 10.3390/nu9111268 29160841 PMC5707740

[27] Carroccio A Mansueto P Iacono G Soresi M D’Alcamo A Cavataio F Non-celiac wheat sensitivity diagnosed by double-blind placebo-controlled challenge: exploring a new clinical entity. *Am J Gastroenterol*. (2012) 107:1898–906. 10.1038/ajg.2012.236 22825366

[28] Catassi C Elli L Bonaz B Bouma G Carroccio A Castillejo G Diagnosis of non-celiac gluten sensitivity (NCGS): the salerno experts’criteria. *Nutrients*. (2015) 7:4966–77. 10.3390/nu7064966 26096570 PMC4488826

[29] Khan A Suarez MG Murray JA. Nonceliac gluten and wheat sensitivity. *Clin Gastroenterol Hepatol.* (2020) 18: 10.1016/j.cgh.2019.04.009 30978535

[30] Molina-Infante J Carroccio A. Suspected nonceliac gluten sensitivity confirmed in few patients after gluten challenge in double-blind, placebo-controlled trials. *Clin Gastroenterol Hepatol.* (2017) 15:339–48. 10.1016/j.cgh.2016.08.007 27523634

[31] Carroccio A D’Alcamo A Iacono G Soresi M Iacobucci R Arini A Persistence of nonceliac wheat sensitivity, based on long-term follow-up. *Gastroenterology.* (2017) 153:56–8.e3. 10.1053/j.gastro.2017.03.034 28365444

[32] Carroccio A Soresi M Chiavetta M La Blasca F Compagnoni S Giuliano A Frequency and clinical aspects of neurological and psychiatric symptoms in patients with non-celiac wheat sensitivity. *Nutrients*. (2021) 13:1971. 10.3390/nu13061971 34201313 PMC8227001

[33] Priyanka P Gayam S Kupec JT. The role of a low fermentable oligosaccharides, disaccharides, monosaccharides, and polyol diet in nonceliac gluten sensitivity. *Gastroenterol Res Pract*. (2018) 2018:1561476. 10.1155/2018/1561476 30158962 PMC6109508

[34] Geisslitz S Longin CFH Koehler P Scherf KA. Comparative quantitative LC-MS/MS analysis of 13 amylase/trypsin inhibitors in ancient and modern Triticum species. *Sci Rep*. (2020) 10:14570. 10.1038/s41598-020-71413-z 32883982 PMC7471314

[35] Ruisi P Ingraffia R Urso V Giambalvo D Alfonzo A Corona O Influence of grain quality, semolinas and baker’s yeast on bread made from old landraces and modern genotypes of Sicilian durum wheat. *Food Res Int*. (2021) 140:110029. 10.1016/j.foodres.2020.110029 33648257

[36] Spisni E Imbesi V Giovanardi E Petrocelli G Alvisi P Valerii MC. Differential physiological responses elicited by ancient and heritage wheat cultivars compared to modern ones. *Nutrients*. (2019) 11:2879. 10.3390/nu11122879 31779167 PMC6950659

[37] Sievers S Rohrbach A Beyer K. Wheat-induced food allergy in childhood: ancient grains seem no way out. *Eur J Nutr.* (2020) 59:2693–707. 10.1007/s00394-019-02116-z 31654113

[38] Geisslitz S Ludwig C Scherf KA Koehler P. Targeted LC-MS/MS reveals similar contents of α-amylase/trypsin-inhibitors as putative triggers of nonceliac gluten sensitivity in all wheat species except einkorn. *J Agric Food Chem*. (2018) 66:12395–403. 10.1021/acs.jafc.8b04411 30365312

