# Supplementary material for: Potential tolerability of ancient grains in non-celiac wheat sensitivity patients: A preliminary evaluation
Source: Front Med (Lausanne). 2022 Sep 28;9:995019. doi: 10.3389/fmed.2022.995019 (PMC9554215; doi:10.3389/fmed.2022.995019)
Supplement: Supplementary file 1 [file Data_Sheet_1.PDF]

Supplementary Material

1. Standard elimination diet and Double-Blind Placebo-Controlled Challenges (DBPCCs)

On entering the study, all patients started a standard elimination diet, which excluded wheat, cow's milk, eggs, tomato and chocolate. Patients self-reporting multiple food hypersensitivity were also asked to avoid ingestion of and/or contact with other food(s) causing symptoms. Food diaries were kept during the elimination diet period to assess dietary intake and adherence to the diet. After 4 weeks of elimination diet, DBPCCs were performed, with the reintroduction of a single food at a time. Patients were randomized to receive either the "active food" or the placebo, according to a computer-generated order determined by an observer not involved in the study.

The DBPCCs were performed with sachets of flour coded A or B containing wheat flour or rice flour, respectively. Sachets A or B were given for 2 consecutive weeks, and then, after 1 week of washout, patients received the other sachets for another 2 weeks (cross-over design). If needed, the washout period was extended for a maximum of a further 2 weeks until the symptoms induced by the previous challenge had completely resolved before starting the next challenge. Wheat challenges were performed by administering a daily dose of 80g of flour, which was dissolved and cooked by the patients themselves. Wheat sachets contained 6.5g of gluten, and an estimated 0.3 g of amylase trypsin inhibitors (ATIs), as determined by bioassay (1).

The codes of the sachets were broken only at the end of the study and the investigators did not know their contents during the study period. Challenges for other foods in patients with suspected multiple food hypersensitivity were performed in an open fashion. During the challenge period, the severity of the intestinal symptoms was recorded: patients completed a 100mm visual analog scale (VAS, with 0 representing no symptoms, and 10 intolerable symptoms), which assessed overall symptoms and the specific symptoms they each reported. The challenges were stopped when clinical reactions occurred for at least two consecutive days (increase in VAS score >30, both for irritable bowel syndrome-like symptoms - onset of abdominal discomfort or pain, associated with a change in stool frequency and/or stool appearance - and for extra-intestinal symptoms). Challenges were considered positive if the same symptoms, which had been initially present, reappeared after their disappearance on the elimination diet, and if the VAS score was >30 when compared to any eventual increase determined during the placebo administration.

References

1. Zevallos VF, Raker VK, Maxeiner J, Scholtes P, Steinbrink K, Schuppan D. Dietary wheat amylase trypsin inhibitors exacerbate murine allergic airway inflammation. *Eur J Nutr*. (2019) 58:1507-14. DOI: 10.1007/s00394-018-1681-6
