# Supplementary material for: Potential tolerability of ancient grains in non-celiac wheat sensitivity patients: A preliminary evaluation
Source: Front Med (Lausanne). 2022 Sep 28;9:995019. doi: 10.3389/fmed.2022.995019 (PMC9554215; doi:10.3389/fmed.2022.995019)
Supplement: Supplementary file 2 [file Data_Sheet_2.PDF]

Supplementary Material

2. Modified version of the Pavia/Biagi score

1. How often do you eat modern wheat-containing food? *Often (score 0), Rarely (score 1), Never (continue the questionnaire).*

2. When you eat away from home, do you inform people about your illness to avoid eating modern wheat? *Yes (continue the questionnaire) or No (score is 2).*

3. Do you check the labels of packaged products? *Yes (score 4) or No (score 3)*
